# Bridging gaps in the elimination of deep mycoses: a comparative analysis of mycetoma and chromoblastomycosis control strategies in Rwanda and China

**DOI:** 10.1186/s41182-025-00778-6

**Published:** 2025-08-25

**Authors:** Alieu Sam, Niyibizi Julius, Munawar Harun Koray

**Affiliations:** 1 Ministry of Health, Freetown, Sierra Leone; 2 Rwanda FDA, Kigali City, Rwanda; 3 Upper West Region Health Directorate, Ghana Health Service, Wa, Ghana; 4 Southern Medical University, Guangzhou, China

**Keywords:** Deep mycoses, Mycetoma, Chromoblastomycosis, China, Rwanda

## Abstract

Deep mycoses, largely caused by mycetoma and chromoblastomycosis, is a chronic fungal infection prevalent in tropics and subtropics regions. Deprived populations in these regions are disproportionately affected. However, control strategies for deep mycoses on a global scale remain limited even though these diseases are included in WHO’s roadmap for 2021–2030 NTDs. China and Rwanda, with distinct climates, economic conditions and health systems and policies, are both burdened with this debilitating fungal infection. This review, therefore, focused on providing a cross-case evaluation of the control strategies for mycetoma and chromoblastomycosis in Rwanda and China by assessing their epidemiology, diagnostics, treatment accessibility and policy frameworks in an effort to find actionable strategies for elimination. Evidence in the control strategies in these different countries can help improve the management of deep mycoses. Rwanda can learn more about advanced diagnostic technologies from China, while China may benefit from adapting the community-based approach from Rwanda. Enhancing surveillance, declaring mycetoma as notifiable disease, and encouraging international cooperation are essential for meeting the WHO 2030 NTD elimination targets.

## Introduction

Deep mycoses are chronic fungal infections classified as neglected tropical diseases (NTDs). It includes mycetoma and chromoblastomycosis (CBM), that affects some of the most marginalized populations living in tropical and subtropical areas, especially with inadequate resources [1, 2]. These devastating infections often lead to progressive tissue destruction causing disability and stigmatization, in addition to poverty and social exclusion [3]. Mycetoma as a neglected tropical disease is estimated to affects approximately 8763 persons globally and a yearly infection of 127 cases of 2013, with an incidence of < 0.01–1.8 cases per 100,000 inhabitants, with Sudan having the highest at 1.8 cases per 100,000 population [4]. The high burden of mycetoma in Sudan was also supported by the report of Zijlstra et al., 2016, stating that over 7000 patients were been treated at the Mycetoma research center in Khartoum, with prevalence in endemic villages reaching 8.5 cases per 1000 (850 cases per 100,000) inhabitants in specific areas [2]. The high burden among rural population is primarily due to their exposure to contaminated soils without protective gears [2]. The global epidemiology of chromoblastomycosis (CBM) from 1914 to 2020 documented 7740 cases globally, excluding Antarctica [3]. Of these figures, South America account for the highest at (2619) cases, followed by Africa (1875) cases, and central America and Mexico (1628) cases, Asia (1390) cases, Oceania (168) cases, Europe (35) cases, and USA and Canada accounting for (25) cases, respectively (3). The burden of the infection predominantly affected the male population at 4022 cases, (81.7%), as opposed to the female population who only suffered 896 infection cases (18.3%), a median age of 52.5 years, an average diagnostic delay of 9.2 years (range 1 month to 50 years) [3]. *Fonsecaea spp.* was responsible for most of the infection as 3089 (80.9%) patients were infected by it, followed by *Cladophialophora spp.* 552 (14.5%), and *Phialophora spp*. 56, (1.5%) [3].

The global burden of deep mycoses is unknown and importantly, the availability of data to guide epidemiology and mortality is limited due to inadequate access to diagnostic capacity and therapeutics [5]. The public health impacts of mycetoma and CBM in the world cannot be understated, yet there is lack of global funding and research, which limit effective fungal control and elimination [5]. The burden of fungal infection is concerning especially for low- and middle-income countries (LMICs), where there is inadequate access to diagnostics and treatment facilities [6, 7]. Recently, the WHO prioritized mycetoma as an NTD, and chromoblastomycosis listed as a fungal priority pathogen, demonstrating a growing burden, estimated to be affecting millions of people across Africa, Asia, and Latin America [8, 9].

Mycetoma is caused by a number of different fungal pathogens, including *Madurella spp* for mycetoma, whereas chromoblastomycosis is associated with dematiaceous fungi, such as *Fonsecaea spp* [9, 10]. Both mycetoma and chromoblastomycosis have similar clinical outcomes as they mimic other diseases like tuberculosis and require molecular diagnostics not commonly available in rural hospitals where their infection rate is high [11]. Treatment for mycetoma and chromoblastomycosis disease is very expensive and take longer time duration to completely heal using antifungal agents [10, 12, 13]. Due to these barriers, foot amputation is common in late stages of treatment [1, 2]. As an NTD, CBM continues to serve as a significant burden in the field of dermatology, particularly in the more severe clinical presentations. Greater efforts to further promote direct microscopy as a basic yet adequate mode of diagnosis for CBM would enhance its diagnosis and treatment especially in rural hospitals [14].

The WHO provided targets for CBM under their 2021–2030 Road Map for NTD; however, little has been done to warrant the initiation or implementation of an effective global control program for CBM. The absence of guiding policy, alongside periods of inactivity, has resulted in the establishment of a Global Chromoblastomycosis Working Group that has suggested a global control strategy for chromoblastomycosis [5]. The global pandemic, COVID-19, has disrupted NTD efforts, with lower funding rates now reported for diseases like mycetoma and chromoblastomycosis, leading to increased inequities [6]. However, the epidemiological burden of these diseases is difficult to determine due to underreporting [8, 9].

The endemicity of mycetoma is evident in the sub-Saharan Africa, with Rwanda having its first two case of actinomycetoma recorded in 2023. Both cases were linked to agricultural exposure and diagnostic delay [15]. Chromoblastomycosis, which is more common in humid regions of tropical Asia and Latin America, and has been recently reported in multiple African countries, including Rwanda and this prevalence was linked to climate change and farming activities [16, 17]. Rwanda and China provide contrasting settings and facilitate unique to the evaluation of scalable solutions for deep mycoses. Rwanda’s community-based health system has made considerable progress in NTDs; however, deep mycoses remain underdiagnosed because of limited fungal expertise in the rural health facilities, reliance on surgical management, and no national policy targeted specifically to these diseases [15, 18]. China’s strong surveillance and diagnostics capacities enhance the early detection of both mycetoma and chromoblastomycosis as opposed to the situation in Rwanda [19].

This review compares mycetoma and chromoblastomycosis control strategies in Rwanda and China, by identifying potentially transferable approaches to elimination through applications of epidemiology, diagnosis, treatment access, and policy.

## Methods

This review used peer-reviewed articles, clinical case reports, and public health policy documents retrieved from multiple databases such as PubMed, ScienceDirect, MEDLINE, Google Scholar, and Web of Science. The following search terms: mycetoma, chromoblastomycosis, deep mycoses, Rwanda, China, neglected tropical diseases, control strategies, diagnosis, treatment, surveillance, and public health were used. The search terms were combined using Boolean operators such as “AND”, and “OR” to refine results. To avoid leaving any gaps in the study, the references’ citations in retrieved articles were listed, and specific searches were conducted in local journals like African Journals Online (AJOL) for data directly related to Rwanda.

A narrative review was performed to compare the two control strategies of mycetoma and chromoblastomycosis in Rwanda and China. The following inclusion criteria were applied: (1) mycetoma or chromoblastomycosis studies conducted in Rwanda, China, or globally which were conducted from 2005 to 2025; (2) English-language peer-reviewed articles, policy documents and case reports related to epidemiology, diagnostic, treatment, policy. Non-peer-reviewed publications and studies, which were irrelevant to these diseases or regions, were excluded. Data were compiled qualitatively, using themes (epidemiology, diagnostics, treatment, policy) to compare approaches.

### Global burden of mycetoma and chromoblastomycosis

Mycetoma and chromoblastomycosis pose important public health problems in tropical and subtropical countries where geographical and occupational conditions increase risk. Mycetoma, which has a chronic subcutaneous course due to infection by fungi or actinomycetes, is found in Africa, South America, and Asia. It is mostly prevalent in Sudan, India and Mexico [20]. Skin lesions of mycetoma and chromoblastomycosis and their disfiguring nature makes it more common in wetter areas like Central and South America, Africa, and Southeast Asia [3]. Both of these diseases affect poor people disproportionately, and suffering from chronic mycetoma leads to severe disability, social stigma and economic burden [21]. The absence of low-cost diagnostics and basic treatment options increases the devastating impact of the disease because there is often no effective intervention before the damage is done, resulting in cruel loss of body tissue and life quality [22].

### Rwanda’s situation of mycetoma and chromoblastomycosis

In Rwanda, the burden of deep mycoses like mycetoma and chromoblastomycosis remains greatly neglected due to poor reporting, and insufficient capacity for diagnosing them [23]. The first documented case of abdominal actinomycetoma in 2023 exemplifies the difficulties in identifying such infections as it was mismanaged to be thought of as an abdominal wall abscess [15]. This highlights a more deep-seated challenge such as lack of adequate specialized diagnostic equipment and sufficiently trained personnel results in ignored advanced cases which leads to misdiagnosis, and prolonged treatment [23]. Contaminated soil, combined with lack of protective footwear, and agricultural work are some of the significant risk factors these rural populations face in Rwanda, which coupled with traditional healing methods and a reluctance to seek care pose a deep-seated challenge to effective disease mitigation [23].

The lack of a national surveillance system in Rwanda for fungal infections, and epidemiological data availability, leads to unreliable estimates of prevalence [24]. For instance, it was only very recently that a single hospital in Kigali was able to perform fungal culture or smears. Until 2024, there had effectively been no lab in Rwanda with that capacity [23]. Emerging fungal infections such as *Emergomyces spp*. and *Blastomyces percursus* also suggest that a more widespread prevalence exists than was previously reported; however, diagnostic limitations exist to determine how prevalent they are [23]. The absence of national policy infrastructure on neglected tropical diseases (NTDs) aggravates these challenges and leaves health care providers to manage mycetoma and chromoblastomycosis without a roadmap or evidence-based resource.

### Overview of China’s situation on the diagnosis, management, documentation, research and surveillances of mycetoma and chromoblastomycosis

China has recorded many cases of chromoblastomycosis and mycetoma cases due to the updating of diagnostics and reporting systems [25]. A retrospective study in Southern China collected 73 chromoblastomycosis cases, primarily due to *Fonsecaea pedrosoi*, with a male-to-female ratio of 4:1 and an average age of 55 years [25, 26]. Similarly, a 10-year study in various provinces of China collected 128 chromoblastomycosis cases, with *Fonsecaea monophora* outlining some of the different pathogens illustrating where chromoblastomycosis was diagnosed [27–31]. In Eastern China cases of *Cladophialophora carrionii* were diagnosed, and PCR confirmed molecular identification [32, 33].

A global study included data from China and reported that Fonsecaea species accounted for 70% of humid region cases of chromoblastomycosis [13, 34]. For mycetoma, a 5-year multicenter study reported 45 cases with Actinomadura madurae as the causative agent in most cases, affecting rural farmers [35]. A case series from Western China reported 12 mycetoma cases and noted the increase of mycetoma cases from arid regions [26, 27, 31]. While rural provinces have seen increased detection of cases, a 2022 study reported 28 cases of diagnosis mycetoma by PCR highlighting easier access to diagnostic assessments [30, 34]. Based on the epidemiology trends, the reported cases are an upward trend, with estimates of 1 case of chromoblastomycosis per 100,000 populations as endemic as low as 1 case, with mycetoma as low as 0.5 cases per 100,000 [14, 30, 36, 37]. These estimates are reflective of China’s ability to accumulate cases regularly for reporting and attributed to the expansion of inexpensive diagnostic assessments available as well as increased awareness [14, 30, 35].

China’s surveillance programs for deep mycoses have a hospital-based network, dermatology center network, and health program surveillance and identification levels to ascertain cases [25]. A 5-year multicenter study from southern China reported the ongoing surveillance for chromoblastomycosis and mycetoma through dermatology centers when dermatology clinics from each institutions ensured hospitals reported chromoblastomycosis in ten hospitals and identified 92 cases [30]. Hospital-based surveillance programs have standardized the reporting of these infections particularly in urban centers, where 85% of fungal infections including subcutaneous mycoses were opportunity to obtain retrospective data from recent electronic health records [27, 30, 31, 38]. In rural areas there is access to limited health care which limits the capacity of health care systems to report complaints of chromoblastomycosis [14, 25].

Further, if there are no health records and 60% of an author’s reports of chromoblastomycosis from remote provinces timeliness or confirmation of diagnosis would be unpredictable [26]. Surveillance through dermatology networks have been impactful by mapping patterns of the subcutaneous mycoses cases by network as indicated by study deductive of 30% move of mapping the 2018–2022 observations of subcutaneous mycoses cases, through the network over 15 provinces (Li et al., 2023). In terms of improving rural surveillance programs mobile health units improved the timeliness of reporting mycetoma cases by recognizing a 25% increase in reports of mycetoma cases documenting the spectrum of cases throughout rural regions with rural health programs connected by PCR [13, 14, 26, 39, 40]. These systems are able to compare with global systems, but the challenge of underreported rates is not uncommon. Importantly, although health care systems are generally recognized as projects, China’s health ministry is important and can offer an opportunity to explain its contributions and investments in infrastructure to provide global health counts for monitoring neglected diseases [39].

China has been leading the way in research on chromoblastomycosis and mycetoma during the past decade, concentrating on molecular diagnostics, antifungal therapy, and genomics [3]. Molecular diagnostics have rapidly evolved with the development of PCR-based identification, also known as molecular typing, which has decreased the time frame of diagnosis by 40% in patients with mycetoma [25, 30, 39]. In addition, another study reported on real-time PCR assays for chromoblastomycosis, achieving an impressive 95% sensitivity rate when detecting Fonsecaea species [30].

### Current control strategies

China has made great effort in the fight against neglected tropical diseases, such as deep mycoses (i.e., mycetoma and chromoblastomycosis) by aligning their national health policies with global strategies [9]. The elimination of lymphatic filariasis and trachoma in 2007 and 2015, respectively, show case China’s commitment to the global discourse through implementing a strong public health framework which could be adapted for mycetoma and chromoblastomycosis [41]. The formation of the China Fungal Disease Surveillance System (CFDSS) in 2025 marks a turning point in the fight against fungal diseases such as chromoblastomycosis, by putting it as a component in the surveillance, prevention, and treatment as part of national health program, shows China’s move in the global fight against fungal diseases [42].

Although mycetoma is not explicitly mentioned in the CFDSS, its inclusion in the WHO’s NTD roadmap (2021–2030) suggests potential for integration into China’s broader NTD programs, particularly given the roadmap’s emphasis on skin NTDs [43]. Lessons learned from China’s strategies used in the control of schistosomiasis enhances integrated approach and professional agencies which will offer an intersectoral collaboration and socio-economics development initiatives that can be useful in the coordination of mycetoma and chromoblastomycosis control [44]. The exclusion of mycetoma from the list of notifiable diseases highlights a major policy gap, which in turn limit proper resource allocation and weakens efforts to control the disease [7].

### Surveillance and diagnostic tools

The establishment of CFDSS has strengthened China’s surveillance system for NTD through its capacity to provide a three-tier monitoring structure for fungal diseases country wide [42]. Evidence suggests that chromoblastomycosis caused by *Cladophialophora carrionii and Fonsecaea* species falls within the scope of the program, as it focuses specifically on fungal pathogens [19]. However, the status of surveillance on mycetoma is uncertain as it is not specific to notification, and this could render trends difficult to ascertain [45].

While diagnostic capabilities and molecular techniques with PCR have improved significantly in China, additional tests using next-generation sequencing (NGS) increase the efficiency in opportunistic fungi and recognition of respective fungal species in cases involving subcutaneous mycoses and invasive fungal diseases [39, 46], all may be applicable to examining chromoblastomycosis and mycetoma cases relating to Sporothrix schenckii, etc. [39, 46]. In low resource setting, the availability of advanced diagnostic tools like PCR and Next Generation Sequencing remains a significant challenge. Conventional microscopy and fungal cultures is the standard for diagnosing mycetoma and chromoblastomycosis [4]. These basic mycology techniques are relatively easy to perform and affordable as they can provide identification of typical fungal grains associated with mycetoma or muriform cells linked with chromoblastomycosis with very little infrastructure [23]. The dependence on these methodologies often results in Epstein–Barr virus (EBV) associated lymphoproliferative disease that are misdiagnosed and attributed owing to absence of specialized personnel or specific mycological skills, limited diagnostic capabilities among clinical personnel, poor awareness about deep mycoses [1, 23]. In low- and middle-income regions like Africa where there is scarcity in mental health trained clinicians, misdiagnosing these infections as tuberculosis or bacterial is often at high chances due to the abscess because of similar signs and symptoms [2]. Improving institutional policies focusing on enhancing laboratory technician training, and understanding the local epidemiology of the disease can significantly improve diagnostic reliability in fragmented healthcare systems until advanced technologies become widely available [47].

### Treatment protocols and healthcare infrastructure

The combination of physical therapy like surgery or photodynamic with itraconazole and terbinafine as primary agents for chromoblastomycosis and mycetoma treatment protocols emphasizes on systemic antifungal therapy being the focus of fungal treatment in China [39]. The establishment of technical guideline by the CFDSS for the rational use of antifungal drugs is essential in reducing the risk of resistance [42]. Although there is a significant improvement in China’s healthcare infrastructure especially in urban cities where there are specialized facilities, there is the need for more attention in eliminating the barriers of limited access to healthcare and high cost of treatment in rural areas [47]. The lack of pharmacovigilance system not been tailored to NTD overlook the adverse drug reaction in mycetoma and chromoblastomycosis treatment [48]. Improvement in water and hygiene sanitation (WASH) also play key role in reducing relapse risk through eliminating underlying risk factors for deep mycoses [39, 41].

### Rwanda’s efforts

#### Existing public health policies

Rwanda national health framework for NTD control program has noted significant success in the fight against schistosomiasis and other fungi diseases, thereby positioning it as a leader in the elimination effort [24, 49]. Rwanda’s effort in aligning with WHO NTD roadmap (2021–2030) through integrating management of skin NTDs such as mycetoma and chromoblastomycosis, even though they are not priorities in many national policies [43]. The adoption of one health approach in schistosomiasis control program depicts a great chance for mycetoma and chromoblastomycosis into the existing frame works, enhancing water sanitation and hygiene intervention which could help control environmental risk factors of the diseases [24]. The high rate of poverty, couple with other contributing diseases and limited infrastructure especially in rural areas greatly impedes policy implementation, thus require targeted intervention for equitable access to care [22]. The inability of Rwanda’s health sector listing mycetoma as a notifiable disease further complicate the fight against it.

#### Access to diagnosis and treatment

The next-generation sequencing (NGS), molecular diagnostics, and other sophisticated tools are not readily available in Rwanda. This makes diagnosing deep fungal infections like mycetoma and chromoblastomycosis apply cost-effective microscopy and histopathology [23]. The gross unavailability of these diagnostic capacity in rural areas where other healthcare infrastructures is also lacking has exacerbated the burden of the diseases [48]. Lack of trained mycologists coupled with scarce technical expertise, low clinical knowledge, and slow equipment purchasing processes, often leads to misdiagnosis of infections that resemble tuberculosis and bacterial abscesses [11]. Due to these barriers, treatment options are significantly limited with high cost of antifungals like itraconazole, and other surgical instrument for mycetoma and chromoblastomycosis management [23]. Most of these infections are left untreated due to their odd resemblance with more common ailments [1, 2]. Risk of treatment toxicity is very high among patient due to pharmacovigilance system not being focus on NTDs [48]. To bridge these gaps, Rwanda can strengthen diagnostic capacity by enhancing training for lab technicians and clinicians, developing affordable point-of-care molecular tools like LAMP, streamlining procurement to reduce red tape, and raising awareness about these diseases through community and healthcare campaigns [50]. The use of community health workers in scaling up malaria and schistosomiasis through mass drug administration give hope for improving the antifungal treatment situation in Rwanda, this can be achieved through investing in the health infrastructure and providing training for healthcare workers [24, 51]. These measures will enhance the use of local resources combined with international expertise to increase diagnostics accuracy while maintaining treatment access for patient across all sector of healthcare.

#### Role of international partnerships

The international effort has been great in the fight against NTD in Rwanda, through disease mapping, mass drug administration and capacity building by funds provided by END fund and the World Health Organization WHO [22, 49]. The presence of these collaboration is hugely felt in Rwanda schistosomiasis program, which could be extended to mycetoma and chromoblastomycosis through training programs and provision of resources, which is in line with China’s intent collaboration with Africa countries for schistosomiasis control [44].

Multi-sectoral collaboration with academic institutions, NGOs and other line ministries have contributed to rapid improvement in Rwanda’s diagnostic and surveillance capabilities, as evident in the malaria control, but gap still remain in the availability of fungal diseases expert [23, 51]. WHO 2020 report on NTD roadmap encourages cross-sectoral collaboration, which Rwanda could take opportunity of by securing funding and technical support for mycetoma and chromoblastomycosis which will boast the country’s ownership of the fungal program [22, 43].

#### Comparative analysis

The presence of advance surveillance system in China couple with molecular diagnostics and a robust healthcare infrastructure used in the fight against mycetoma and chromoblastomycosis is a significant mile stone in fungal disease elimination [42, 46]. Diagnosis and treatment access for mycetoma and chromoblastomycosis is limited in Rwanda, but there has been great improvement in integrating NTD control into the national health system and enhancing international partnership offering replicable model for resource constrains settings [24, 49]. Both China and Rwanda face challenges in enhancing healthcare access for the rural communities, and lack of putting mycetoma as a notifiable disease hinders effective surveillance and resource allocation for this neglected tropical disease (NTD’s) [7, 45].

China on the other hand employs an advanced diagnostic capability, such as ITS rDNA sequencing, and innovative therapies, such as itraconazole–terbinafine combination therapy and photodynamic therapy with a 68% improvement rate for chromoblastomycosis in provinces like Guangdong [14, 30]. At the same time, in China, there is no singular unified national strategy, endless gaps in rural access, and inequitable care still exist [14, 30]. Although health systems in China have progressed, there is still no coordinated national strategy, and access and equity continue to be challenges in rural areas of the country leading to misdiagnosed of Chromoblastomycosis as other diseases [14]. Comparisons between Rwanda and China showed how the factors, and priority across health systems leads to differences in actual outcomes in NTDs control and the mitigation of deep mycoses.

The strong advancement in China’s socioeconomic, technological, policy and education provide a better foundation for addressing disease burdens and environmental risk factors, whereas, Rwanda’s over reliance on external funding poses sustainability risk and impedes national coordination for disease control [22, 41]. Among the many sophisticated technology in China, adopting Rwanda’s community-based model with their diagnostics and surveillance expertise could bridge the gap in deep mycoses control, which will not only align with global target for NTD elimination by 2030, but will also enhance rural population’s access to healthcare at all levels [43].

## Conclusion

This comparative research study of mycetoma and chromoblastomycosis control strategies in China and Rwanda identifies string unique strengths and shared difficulties related to the deep mycoses by demonstrating the value of both parallel and specialized learning in both countries. China’s diagnostic infrastructure, particularly use of PCR and next-generation sequencing, improved case detection in clinical-chromoblastomycosis areas like Guangdong province, which responded positively to combination therapies. In contrast, Rwanda’s community health policies and international collaborations have advanced NTD control, but lack and poor of mycological knowledge and diagnostic facilities results in underdiagnosis and surgical treatment. Both countries are still challenged by restricted access to healthcare services in rural area coverages, the absence of mycetoma as a notifiable disease which limits effective surveillance systems, and poor resource allocation.

The results emphasize the need for targeted models. China’s molecular diagnostics and surveillance frameworks could be helpful to Rwanda if adapted to fit their lacks of resources. Contrarily, China could learn from Rwanda’s model of community health workers (CHW’s) to improve and strengthen outreach services in rural areas. These policies should consider mycetoma as a notable disease, increasing supervision over antifungal therapy side effects, and fortifying water, sanitation and hygiene measures to reduce environment related risk factors. More work is needed to develop economical diagnostic tools, scalable treatment regimens, and epidemiological studies focused on underreported data most especially in Rwanda where data are scarce. Collaborative effort is critical, as the WHO NTD roadmap for 2021–2030 advocates, supporting knowledge sharing and resource mobilization. Along with these findings, the two countries can work towards the elimination of deep mycoses, in balance with sustainable development goals and the alleviation of burden on marginalized populations.

## Data Availability

No datasets were generated or analyzed during the current study.

## Abbreviations

NTDs: Neglected tropical diseases
CBM: Chromoblastomycosis
WHO: World Health Organization
LMICs: Low- and middle-income countries
CFDSS: China Fungal Disease Surveillance System
PCR: Polymerase chain reaction
NGS: Next Generation Sequence
WASH: Water, Sanitation, and Hygiene
CHWs: Community health workers
END: FUND End Neglected Tropical Disease Fund
ITS r DNA: Internal transcribed spacer ribosomal deoxyribonucleic acid

## Declarations

### Protocols and registry

This review does not require registry as no protocols was established.
